# Stem Shading Promotes Mannitol Accumulation in the Bark of *Fraxinus ornus* and Prevents Sucrose Transport in Roots Under Drought

**DOI:** 10.1111/ppl.70894

**Published:** 2026-04-20

**Authors:** Sara Gargiulo, Sara Natale, Alessandro Pichierri, Martina Tomasella, Francesco Petruzzellis, Andrea Nardini, Valentino Casolo

**Affiliations:** ^1^ Department of Agri‐Culture, Food, Environmental and Animals Sciences University of Udine Udine Italy; ^2^ Department of Biology University of Padua Padova Italy; ^3^ National Biodiversity Future Centre Palermo Italy; ^4^ VCR Research Center San Giorgio della Richinvelda Italy; ^5^ Department of Life Sciences University of Trieste Trieste Italy

**Keywords:** energetic, hydraulics, stem photosynthesis, sugars, woody plants

## Abstract

Drought induces the accumulation of osmolytes, including soluble non‐structural carbohydrates (NSC), to support osmotic adjustment and hydraulic recovery. Bark has been proposed as a major site of sugar storage, with stem photosynthesis potentially contributing to NSC production and drought responses. Some species also accumulate polyols, such as mannitol, often linked to membrane protection. However, the allocation of carbon compounds among plant organs during drought and recovery remains unclear. In this study, *Fraxinus ornus* saplings were exposed to stem shading, followed by drought and recovery to investigate the single and combined effects of these factors on NSC allocation in bark, wood, and roots. Hydraulic parameters were measured alongside concentrations of glucose, fructose, sucrose, starch, and mannitol under well‐watered, drought, and recovery conditions. Stem shading increased xylem vulnerability to embolism and reduced glucose concentration in stems and roots, while other sugars and mannitol were unaffected. Drought triggered starch degradation and increased hydraulic conductance loss, regardless of light treatment. Sucrose concentration increased in bark and roots, especially in non‐shaded plants, whereas mannitol increased mainly under combined drought and shading. During recovery, sucrose declined, whereas mannitol remained elevated. Our results indicate that carbon partitioning in *F. ornus* is strongly affected by drought and influenced by stem shading. Root sucrose appears central to whole‐plant osmotic adjustment but is sensitive to shading. Mannitol, likely sustained by starch degradation, may instead support osmotic adjustment during recovery, representing a more carbon‐efficient osmolyte.

## 1 Introduction

In recent decades, woodlands have been increasingly affected by rising air temperatures and altered rainfall regimes, resulting in widespread crown die‐back and increased tree mortality across different forest ecosystems (Rodman et al. 2025). Drought‐induced tree mortality is primarily associated with hydraulic failure, often occurring in combination with carbon starvation (McDowell et al. 2022), as water deficit limits photosynthesis and carbohydrate transport. Low tissue water potential during drought can further impair cellular functionality and the translocation of soluble non‐structural carbohydrates (NSC) and other secondary metabolites involved in stress responses (Raza et al. 2025).

Consequently, the comprehension of how plants cope with drought and the functional features critical to survive or succumb to extreme climatic events requires the understanding of dynamics, flows, and regulation of water and NSC pools in different plant organs (Hubeau and Steppe 2015).

Water deficit induces stomatal closure and reduces carbon uptake, making tree survival also dependent on the capacity to mobilize and utilize NSC reserves (Jupa et al. 2024). Under stress conditions, long‐term reserves such as starch can be hydrolysed to meet energy demand and contribute to osmotic adjustment (Tomasella, Petrussa, et al. 2019). In woody plants, sugar translocation occurs both via apoplastic phloem loading, mediated by sucrose transporters (Xu et al. 2018), and symplastic loading through a sucrose concentration gradient between source and sink (Braun 2022).

In this context, stem photosynthesis in woody plants has gained attention as a potential contributor to plant carbon balance under stress. Stem photosynthesis occurs in both gymnosperms and angiosperms (Pfanz et al. 2002) and can provide an additional carbon source to sustain plant metabolism, especially when leaf photosynthesis is constrained, such as during drought (Vandegehuchte et al. 2015) or because of leaf loss (Kocurek et al. 2025). While leaf photosynthesis is fuelled by atmospheric CO_2_, woody tissues can also exploit endogenous CO_2_ produced during respiration to sustain photosynthesis and exhibit higher water‐use efficiency due to lower peridermal water vapour conductance, making stem photosynthesis less sensitive to water deficit (Wittmann and Pfanz 2008). Moreover, bark tissues in young, photosynthetically active stems represent a substantial proportion of stem volume (Rosell et al. 2017), allowing a significant NSC storage capacity (Rosell et al. 2021), which is 1.5–9‐fold higher than wood in terms of concentration (Wiley et al. 2019). These observations have led to the hypothesis that the inhibition of stem photosynthesis may jeopardize local NSC availability, thereby reducing osmotic adjustment capacity, compromising embolism repair mechanisms, and limiting stem growth (Kocurek et al. 2020; Ávila‐Lovera et al. 2024) during drought and recovery phases. In this perspective, the direct effect of stem shading on carbon metabolism warrants specific consideration. By reducing irradiance on photosynthetically active bark tissues, shading lowers gross stem photosynthesis and limits the reassimilation of internally respired CO_2_ (Pfanz et al. 2002). This decrease in local carbon gain may alter the balance between sucrose synthesis, export, and transient starch accumulation, thereby modifying NSC partitioning within the stem. Because carbon supply is tightly linked to growth processes, shading can also influence biomass accumulation and allocation patterns, potentially reducing radial growth and structural investment in woody tissues (Wang and Wang 2023).

Besides NSC, plants accumulate compatible osmolytes, such as amino acids, polyamines, and sugar alcohols (polyols), in the cytoplasm to maintain cell turgor (Ozturk et al. 2021). Studies on conifers reported pinitol accumulation in woody tissues during frost desiccation (Deslauriers et al. 2014) and during summer drought (Tomasella et al. 2017). Other studies on *Prunus* spp. evidenced sorbitol increase in leaves and fruits in response to drought (Lo Bianco et al. 2003; Jiménez et al. 2013). However, the contribution of polyols to drought tolerance in trees remains insufficiently understood, particularly at the whole‐plant level.

Mannitol is a sugar alcohol derived from fructose and commonly produced in representatives of the Oleaceae family, where it has been associated with stress tolerance and oxidative stress mitigation (Patel and Williamson 2016). Although mannitol exerts an osmotic effect comparable to that of hexose sugars and sucrose, it is not directly involved in glycolysis, suggesting a role primarily as a compatible solute rather than as a metabolic substrate. Indeed, it was proposed that drought induces an increase in mannitol concentration in xylem sap to prompt stomatal closure (Patonnier et al. 1999) and that mannitol production is sustained by starch degradation in woody tissues (Tsamir‐Rimon et al. 2021). Given the close metabolic link between mannitol synthesis and glucose metabolism, the quantification of individual NSC pools becomes essential to fill the gap in understanding the different roles of glucose, sucrose, and mannitol under drought and recovery.

In a previous study by some of us, we demonstrated the presence of functional chloroplasts in the stems of *Fraxinus ornus* (Natale, La Rocca, et al. 2023), underscoring the importance of stem photosynthesis as a source of local NSC contributing to osmoregulation. However, it was not explored which pool of soluble NSC was mostly involved in drought responses (Natale, Tomasella, et al. 2023). Indeed, light conditions affect the interplay between hexoses, sucrose, and transient starch, thereby influencing osmotic potential and limiting the interpretation of plant response based solely on total soluble NSC. Moreover, in the previous study, the potential role of mannitol as an osmolyte was not investigated. However, such a role would be reasonable considering that this molecule is a very representative polyol of Fraxinus species.

Here, we hypothesized that alterations in stem light conditions may affect NSC partitioning during drought stress. To test this hypothesis, we designed a set of experiments aimed at verifying the effect of stem shading on the allocation of the main monosaccharides (glucose and fructose) and disaccharides (maltose and sucrose) to different organs. We expected a reduction in sucrose production due to stem shading, which should prevent or delay post‐drought recovery. Moreover, we hypothesized that mannitol is involved in osmotic adjustment under drought conditions and in hydraulic recovery when sucrose is limited, particularly under stem shading. Mannitol, indeed, may confer several potential advantages over other solutes, including a more efficient carbon use pathway, especially in sink tissues (Stoop et al. 1996). As a compatible solute, mannitol has the same osmotic efficiency as sucrose but can be synthesized from a single glucose‐6‐phosphate molecule rather than two (Patel and Williamson 2016), making it especially advantageous under conditions of limited carbon supply and reduced ATP. Finally, our present study was aimed at analyzing as well as NSC allocation to roots, which are often neglected in carbohydrate analysis.

## 2 Material and Methods

### 2.1 Plant Material

Plant material was the same as that used in a previous experiment on *Fraxinus ornus* L. (for details see Natale, Tomasella, et al. 2023). *F. ornus* is a woody species widely distributed in Southern Europe and frequently investigated in drought stress/recovery experiments, also in combination with light manipulation (Tomasella, Casolo, et al. 2019). This species exhibits a bark chlorenchyma in adult trees (Figure S1) and a recent study reported the presence of functional chloroplasts in bark and wood (Natale, La Rocca, et al. 2023), making this species particularly useful for deepening our understanding of the role of stem photosynthesis in woody plants.

### 2.2 Experimental Design

**FIGURE 1:**
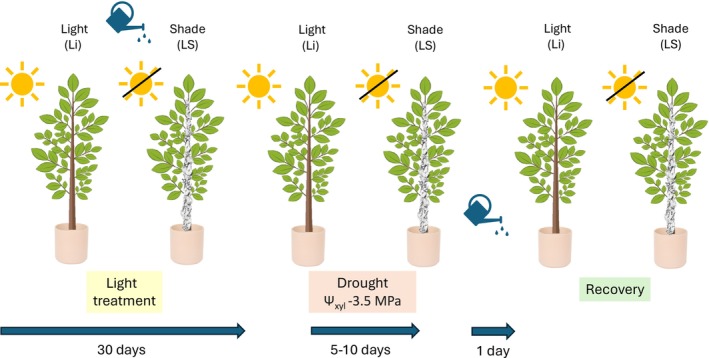
Experimental design. Saplings of *Fraxinus ornus* were fully exposed to light or stem shading for 30 days. All plants were subjected to drought (Ψxyl < −3.5 MPa) and re‐irrigated to assess recovery after 1 day.

Three‐year‐old saplings were transplanted in 3.5 L pots, grown in a greenhouse, and regularly irrigated to the maximum soil water capacity. Throughout the experiment, the temperature was 26.67°C ± 2.17°C and RH 77.48% ± 6.15%. Plants were divided into two groups: lighted plants (Li) always fully exposed to light under natural illumination, and shaded plants (LS) subjected to stem light exclusion for 30 days by covering the stems with loosely wrapped aluminium foils, so that gas exchange was still possible. After this time interval, five plants from each group were kept under well‐watered conditions (control), while the other five plants were exposed to an experimental drought (drought) by suspending irrigation for 5–10 days, until xylem water potential (Ψ_xyl_) dropped below −3.5 MPa, that is, value corresponded to the 50% loss of xylem hydraulic conductance in this species (Petit et al. 2016). Afterwards, drought‐treated plants were re‐irrigated at pot capacity and re‐evaluated for eventual recovery of hydraulic functions (recovery) (Figure 1). Five plants were sampled to measure hydraulic parameters and NSC concentration for each treatment.

### 2.3 Chlorophyll Analysis

In order to verify the effectiveness of stem shading with aluminium foils, chlorophyll *a* and chlorophyll *b* concentrations in bark were quantified. Stem samples were collected and immediately placed in the dark to perform pigment extraction. The stems were decorticated to separate bark and wood. Fresh material of bark samples was cut into small pieces and approx. 50 mg of bark were placed into a 2 mL Eppendorf tube containing 1.5 mL of 80% acetone solution. Absorption spectra were recorded using a UV–Vis spectrophotometer in the wavelength range between 250 and 750 nm. Final chlorophyll concentration was calculated according to the equations proposed by Wellburn (1994).

### 2.4 Leaf Water Potential and Hydraulic Measurements

Midday stem xylem water potential (Ψ_xyl_, MPa) and percentage loss of hydraulic conductance (PLC) data reported in this study are the same as published in Natale, Tomasella, et al. (2023). In brief, to estimate midday stem xylem water potential (Ψ_xyl_, MPa), 1–2 mature leaves were wrapped in cling film and covered with aluminium foil in the early morning. Leaves were then sampled between 11:00 and 14:00 h (solar time), and Ψ_xyl_ was measured using a pressure chamber (mod. 1505D, PMS Instrument Co.) At the same time, light intensity (μmol photons m^2^ s^−1^) was measured with a Quantum photo‐radiometer (HD 9021, Delta OHM S.r.l.) at the selected leaf surface. To investigate whether stem shading influenced the xylem vulnerability to embolism, we measured the percentage loss of hydraulic conductance (PLC) in 1‐year‐old stem segments from Li and LS plants. PLC was measured in controls (well‐watered plants), as well as in drought and recovery treatments. PLC was determined following the approach described previously by Secchi and Zwieniecki (2011). Briefly, small stem segments (4–6 cm long) were cut underwater to prevent embolism formation. Initially, the segments were perfused at a pressure (P) of 0.08 MPa, and the corresponding flow rate (F) was measured to obtain the initial stem hydraulic conductance value (K_i_). Subsequently, the samples were flushed at *p* = 0.15 MPa for 10 min to refill embolized vessels, and F was re‐measured at *p* = 0.08 MPa to obtain the K value after embolism removal (K_max_). PLC was calculated as: PLC = 100 × [1 − (K_i_/K_max_)].

### 2.5 Non‐Structural Carbohydrates

The analysis of NSC was performed on the same plants used for the hydraulic measurements. From each individual, 4 cm long stem segments adjacent to the portion used for hydraulic measurements were collected, and bark and wood were separated. On the same plants, we collected 3–4 segments of coarse secondary roots (each 3–4 cm long, 2–5 mm in diameter). Root segments were taken, excluding fine roots (< 2 mm) and large structural roots. Since roots are major sink and storage organs, they typically contain substantial NSC pools. We targeted this root class because it is ecologically relevant for NSC storage and transport and is generally less temporally labile than fine absorptive roots, while still representing the belowground carbohydrate pool connected to the stem hydraulic pathway (Tomasella, Petrussa, et al. 2019; Kannenberg et al. 2018). Adhering soil was rapidly removed with deionized water and damp paper. After that, stem and root samples were immediately microwaved at 700 W for 3 min to block enzymatic activity and related NSC consumption. Afterward, samples were oven‐dried at 70°C for 24 h and then ground in a ball mill (MM400; Retsch GmbH).

Soluble NSC and starch extraction were performed according to the procedures proposed by Quentin et al. (2015) and Landhäusser et al. (2018), as modified for low amounts of material by Gargiulo et al. (2024). Briefly, 15 ± 1 mg of ground powder were suspended in 80% ethanol solution and incubated in an oven at 80°C for 30 min. After centrifugation (7000 *g*) for 3 min, (with Mikro 120, Hettuch zentrifugen) the extracted solution was separated and collected. This step was repeated, and the pellet was reserved for starch hydrolysis. The extracted solution, containing soluble NSC, was incubated in an oven at 55°C until ethanol was completely evaporated. Then, the crystallized soluble NSC were suspended in a buffer solution (50 mM Tris–HCl at pH 7.5). In this suspension, the single pools of NSC were analysed following the enzymatic method described by Savi et al. (2019) with minor modifications for low amounts of plant material (see details in the following paragraphs). Total soluble NSC were finally calculated as the sum of the individual NSC pools.

#### 2.5.1 Glucose

Glucose concentration was determined using the enzymatic method with hexokinase and glucose‐6‐phosphate dehydrogenase and estimated as NADH. The reaction was performed on ELISA microplates by adding 0.3 U/sample of Hexokinase (from yeast, 9001‐51‐8 Megazyme) and 0.5 U/sample of Glucose‐6‐phosphate dehydrogenase (from *Leuconostoc mesenteroides* 9001‐40‐5 Megazyme) in a buffer solution containing 50 mM Tris–HCl pH 7.5. For 1 mL of Tris–HCl, we added 2.5 μL of 2 M MgCl_2_, 20 μL of 50 mM NADP^+^, and 5 μL of 0.4 M NaATP. After incubation in an oven at 32°C for 20 min, the NADPH produced by the reaction was measured at 340 nm with a spectrophotometer (Victor multi‐plate reader, Perkin‐Elmer). The resulting absorbances were calculated using a glucose standard curve as a reference and expressed as g g^−1^ of dry weight.

#### 2.5.2 Fructose, Sucrose, and Maltose

Fructose, sucrose, and maltose concentrations were quantified as glucose units and converted to concentration (mg mg^−1^ of dry weight).

Fructose determination was performed on ELISA microplates by adding 2.5 U/sample of Phosphoglucose isomerase (ammonium sulfate suspension) (from yeast 9001‐41‐6 Sigma) to convert fructose‐6‐phosphate to glucose‐6‐phosphate. Glucose quantification was performed as described above.

For sucrose determination, an aliquot of extract containing soluble NSC was suspended in acetate buffer (25 mM sodium acetate trihydrate at pH 4.6) to acidify the pH and favour invertase activity. In each sample, 20 U of invertase (ammonium sulfate suspension) (from yeast, 9001‐57‐4 Megazyme) were added, and the sucrose hydrolysis was performed at 55°C for 1 h. Glucose obtained from sucrose hydrolysis was quantified following the same procedure described above.

To quantify maltose, another quantity of extract containing soluble NSC was suspended in acetate buffer (25 mM sodium acetate trihydrate at pH 4.6) to acidify the pH and favour amyloglucosidase activity. In each sample, 15 U of amyloglucosidase (ammonium sulfate suspension) (from *Aspergillus niger*, 9032‐08‐0 Sigma) was added, and the maltose hydrolysis was performed at 70°C for 1 h. Glucose quantification followed the same procedure as described above.

#### 2.5.3 Starch

Starch hydrolysis followed the method proposed by Landhäusser et al. (2018). Pellet coming from the separation of starch and soluble NSC (see the alcohol extraction described above) was suspended in 0.4 M of sodium acetate trihydrate (pH 4.6) and samples were boiled for 1 h to allow starch gelatinization. After cooling at room temperature, 100 U of α‐amylase (from porcine pancreatic, Megazyme) and 25 U of amyloglucosidase (from *Aspergillus niger*, Megazyme) were added to each sample and starch hydrolysis was performed at 55°C in oven overnight. Before the analysis, samples were boiled to stop enzyme activity.

Starch quantification, as well as that of single soluble NSC, was performed by measuring glucose as NADPH (see glucose analysis).

#### 2.5.4 Mannitol

Mannitol concentration was determined enzymatically, according to the method described by Lunn et al. (1989), with slight modifications as follows. In a buffer solution (50 mM Tris–HCl at pH 8), 50 mM NAD^+^ was suspended and 0.6 U/sample of mannitol dehydrogenase added. The reaction was carried out at 40°C and mannitol concentration was quantified as NADH formation with spectrophotometer at 340 nm. The resulting absorbances were compared with known amounts of mannitol (Figure 1).

### 2.6 Data Analysis

All statistical analyses were performed in R (version 4.3.2, R Core Team, 2023).

Differences in NSCs concentration between plant materials (bark, root and wood) and light treatment levels (Li and LS) were tested by linear models (LM) using the “car” R package (Fox et al. 2001) and plotted using “ggplot2” R package (Lüdecke et al. 2021). Specifically, we set plant materials, light treatment levels and their interaction as the predictive variables. We assumed significant results with *p*‐value ≤ 0.05. Model assumptions were checked by diagnostic plots of residuals. Only for models with significant interaction among explanatory variables, pairwise comparisons were performed using *emmeans* function (Tukey test) in “emmeans” R package (Lenth 2018).

The relationship between PLC and Ψ_xyl_ was tested using an LM, setting PLC as the response variable, and Ψ_xyl_, light treatment levels and their interaction as predictive variables. Similarly, we ran two independent LMs, setting PLC as the response variable and soluble NSC or starch, plant materials, and their interaction as the predictive ones. The outputs of these models were plotted using the “plot model” function in the “ggplot2” R package (Lüdecke et al. 2021).

Differences in sucrose and mannitol content among control, drought, and recovery plants and across light treatment levels were calculated for each plant material using LMs, as in NSCs. Pairwise comparisons were performed for significant interactions as explained above.

The relationship between starch and sucrose concentrations in the root was tested using an LM and plotted with the “plot model” function in the “ggplot2” R package (Lüdecke et al. 2021).

Outliers were removed after the “outlier test” in the “emmeans” R package (Lenth 2018).

## 3 Results

### 3.1 Effect of Shading Treatment on Sugars and Mannitol Content in Well‐Watered Conditions

**FIGURE 2:**
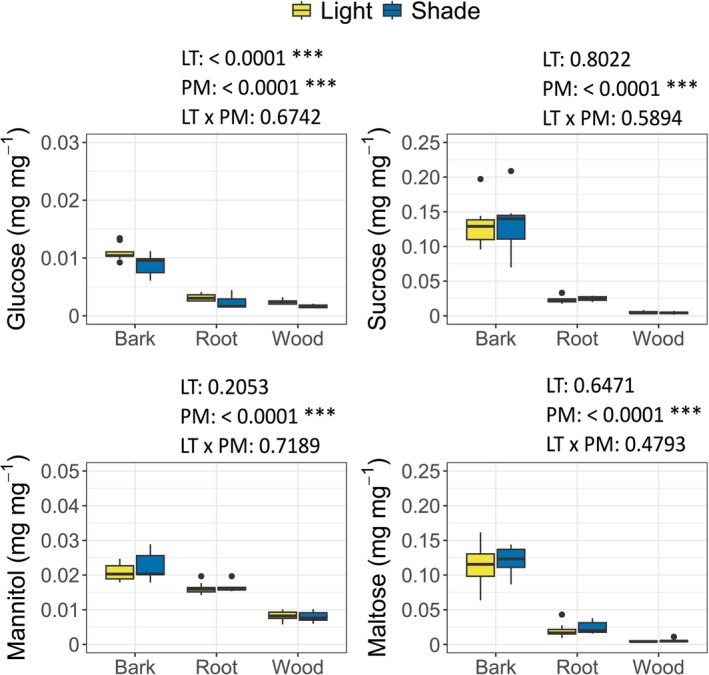
Boxplots of glucose (a), sucrose (b), maltose (c), mannitol (d) concentration in well‐watered conditions after the light treatment in bark, root and wood. Yellow boxes: Lighted plants; blue boxes: Stem‐shaded plants. The effects of the explanatory variables (Light Treatment level, LT; Plant Material, PM) on plant parameters, along with their associated *p*‐values, are also reported. *p < 0.05; **p < 0.01; ***p < 0.001.

Stem shading was applied for 30 days to inhibit stem photosynthesis. We validated the efficacy of the shading by measuring total chlorophyll (*a* + *b*) concentration in bark (Figure S2). As expected, in the bark, the concentration of chlorophyll was lower in the stem‐shaded plants (0.248 ± 0.039 mg g^−1^) compared to the light‐exposed ones (0.400 ± 0.012 mg g^−1^). Figure 2 reports the concentrations of sugars (i.e., glucose, sucrose, maltose) and mannitol in bark, wood, and roots in control Li and LS plants. Fructose is not reported because its concentration was below the limit of detection in all plant materials and treatments.

Across light treatments, sugar and mannitol concentrations differed significantly between plant materials (Table S1). Bark showed the highest concentration of sugars (Figure 2a–c) and mannitol (Figure 2d), followed by roots, whereas wood had the lowest concentration.

Stem‐shading had a limited effect on carbohydrate pools under well‐watered conditions. Between the analysed compounds, only glucose concentration was significantly affected by the shading treatment, showing a consistent reduction in bark, wood, and roots of LS plants compared to Li plants (Figure 2a). In contrast, sucrose, maltose, and mannitol concentrations did not differ between light treatments in any of the plant material.

### 3.2 Relationship Between Percentage Loss of Conductance (PLC) and Xylem Water Potential (Ψ_xyl_)

**FIGURE 3:**
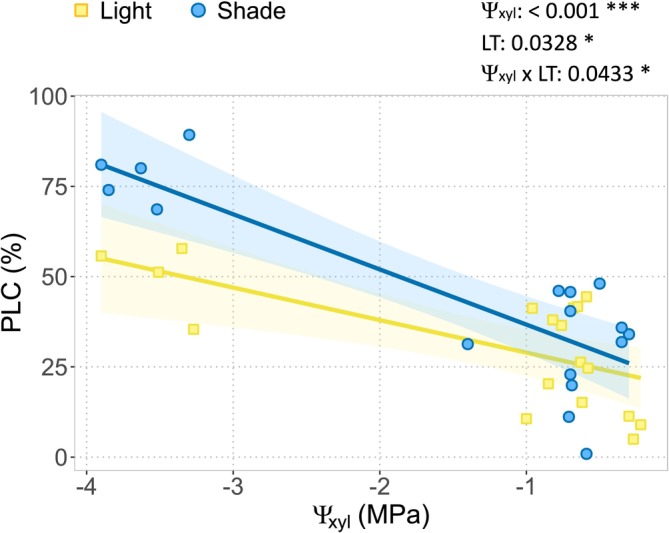
Relationship of percentage loss of hydraulic conductivity (PLC) and xylem water potential (Ψ xyl) in lighted plants (yellow dots) and shaded plants (blue dots), according to the outcomes of ANCOVA. Blue line: Stem‐shaded (LS, *n* = 17) plants; yellow line: Lighted (Li, *n* = 18) plants. Confidence intervals (95%) are shown (light coloured areas). The effects of the explanatory variables (Water Potential, WP; Light Treatment level, LT; and their interaction, WP × LT) on plant parameters and their associated *p*‐values are also reported. *p < 0.05; **p < 0.01; ***p < 0.001.

Across all plants, PLC increased as Ψ_xyl_ became more negative, confirming the expected effect of severe water stress on embolism formation (Figure 3). We detected a higher vulnerability to embolism formation when stem photosynthesis was inhibited. By extrapolating the xylem water potential at 50% loss of hydraulic conductance (P50), we estimated values of −3.4 MPa in lighted plants (consistent with previous observations, for example, Petit et al. 2016) and −1.9 MPa in shaded plants. Consistently, LS plants tended to have higher PLC levels than Li plants (i.e., a statistically significant LT effect; Table S2), and showed a steeper increase in PLC in response to decreasing Ψxyl than Li plants (Figure 3).

### 3.3 Relationship Between Percentage Loss of Conductivity (PLC) and Non‐Structural Carbohydrates (NSC)

**FIGURE 4:**
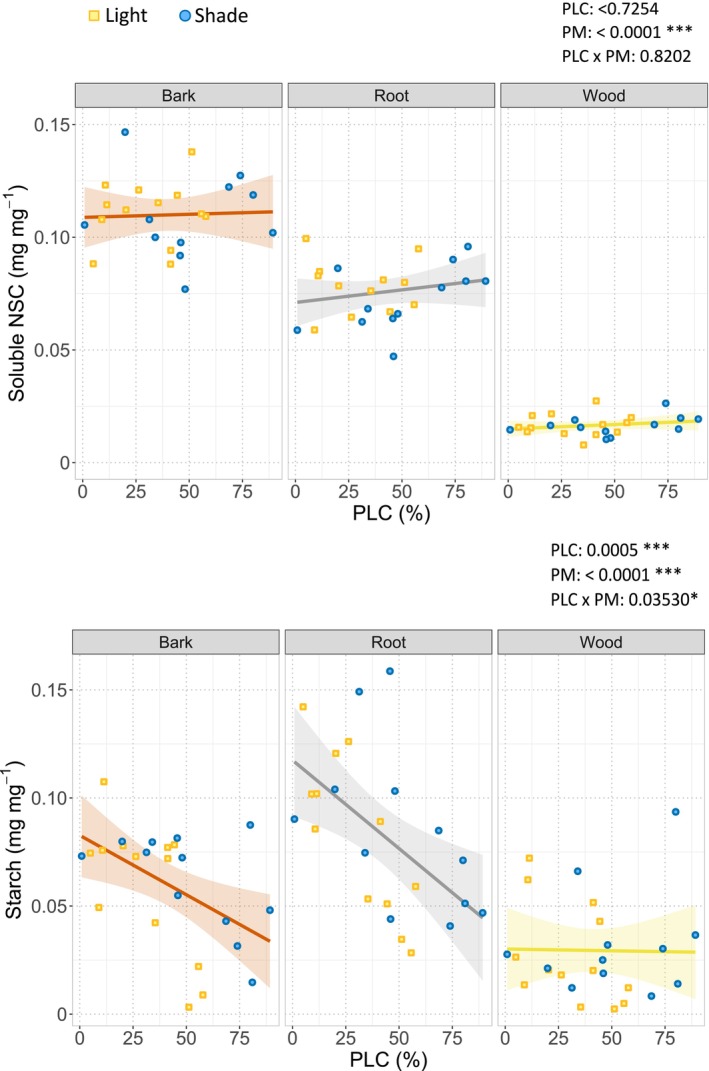
Relationship between soluble NSC (a) and starch (b) concentration in bark, root and wood with the percentage loss of hydraulic conductivity (PLC) of lighted plants (yellow dots, *n* = 18) and shaded plants (blue dots, *n* = 17), according to the outcomes of ANCOVA. Confidence intervals (95%) are shown (light coloured areas). The effects of the explanatory variables (PLC; PM: Plant material; and their interaction, PLC × PM) on NSC and starch concentration and their associated *p*‐values are also reported. *p < 0.05; **p < 0.01; ***p < 0.001.

To assess whether carbon availability was associated with hydraulic failure, relationships between PLC and both total soluble NSC and starch concentrations were analysed in bark, roots, and wood under drought conditions (Table S3). Total soluble NSC concentrations differed markedly among plant materials, with higher values in bark and roots (Figure 4a). However, no significant relationship was observed between total soluble NSC concentration and PLC in any of the plant materials considered. Conversely, starch concentration showed a clearer association with hydraulic impairment (Figure 4b). Stem PLC increased as starch concentration declined in both bark and roots, revealing a significant negative relationship between these variables. This pattern was not observed in the case of stem wood, where starch concentration was low.

### 3.4 Concentration of Sucrose and Mannitol Under Drought and Recovery

Water status significantly affected sucrose and mannitol concentrations in all analysed plant materials (Table S4), but the magnitude and direction of these changes differed between light treatments.

**FIGURE 5:**
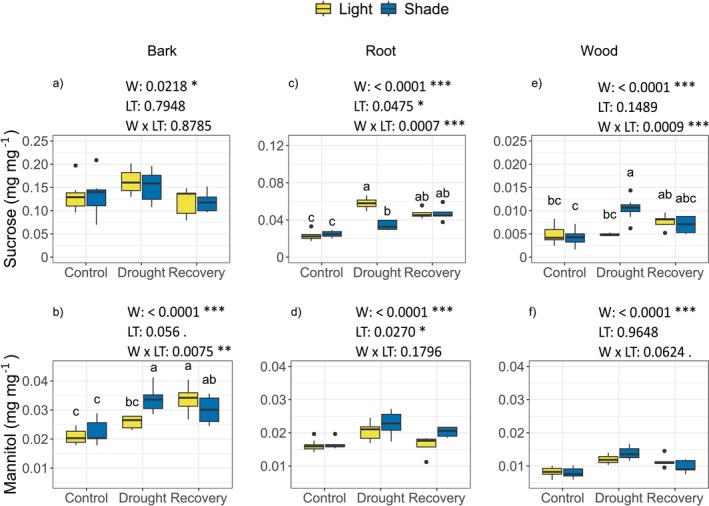
Boxplots of sucrose (a, c and e) and mannitol (b, d and f) concentrations in bark, root and wood along the experiment. Yellow boxes: Lighted (Li) plants; blue boxes: Stem‐shaded (LS) plants. Different letters indicate statically significant differences among treatments (*p* < 0.05). The effects of the explanatory variables (Water, W; Light Treatment, LT; and their interaction, W × LT) on plant parameters and their associated *p*‐values are also reported. *p < 0.05; **p < 0.01; ***p < 0.001.

In bark, sucrose concentration increased during drought. It declined after recovery, without detectable effect of stem shading or interaction between light treatment and water status (Figure 5a). Mannitol, by contrast, showed a strong response to water stress, increasing markedly during drought and remaining elevated during recovery (Figure 5b). On average, mannitol concentration in bark was higher in LS plants than in Li plants, evidencing a marginally significant effect of stem shading. In addition, a significant interaction between light treatment and water status was observed (Figure 5b) in that mannitol concentration increased during drought in LS plants, whereas in Li plants the increase occurred only following the recovery phase.

In roots, stem shading exerted a pronounced effect on both sucrose and mannitol concentration (Figure 5c,d). Under drought, sucrose was significantly lower in LS plants than in Li plants, resulting in a significant interaction between water status and light treatment across the experiment (Figure 5c). This treatment‐dependent behaviour was also reflected in a different relationship between sucrose concentration and PLC in Li and LS plants (Figure S2). In roots, mannitol concentration increased during drought in both treatments. Still, it reached higher values in LS plants, indicating an enhanced accumulation of this polyol when stem photosynthesis was inhibited (Figure 5d). In wood, sucrose and mannitol concentrations were consistently lower compared to bark and roots (Figure 5e,f). Sucrose increased after drought only in LS plants, leading to a significant interaction between water status and light treatment (Figure 5e). Mannitol concentration in wood was affected only by water status, increasing during drought and declining during recovery, with no detectable effect of stem shading (Figure 5f).

### 3.5 Relationship Between Sucrose and Starch in Roots

**FIGURE 6:**
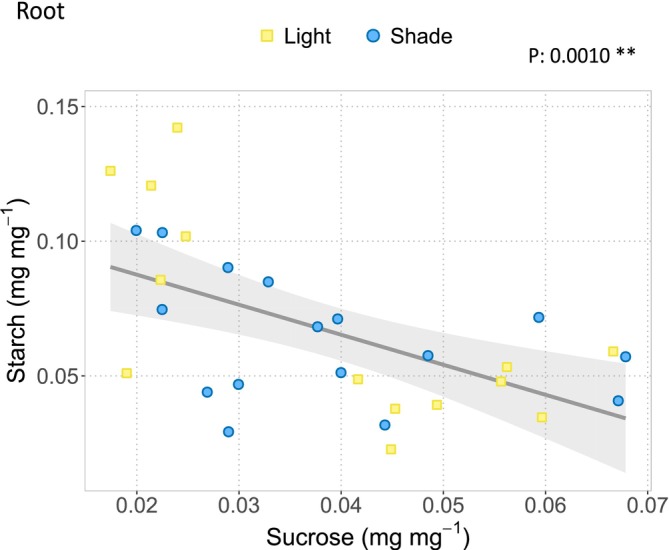
Relationship between starch and sucrose concentrations in root of lighted plants (yellow dots, *n* = 15) and shaded plants (blue dots, *N* = 16). Confidence interval (95%) is shown (blue area). *p*‐value, as obtained by the one‐way ANOVA analysis is reported. *p < 0.05; **p < 0.01; ***p < 0.001.

Given the observed decrease in starch concentration in bark and roots during drought (Figure 4), we examined possible relationships between starch and sucrose concentrations across the different compartments. A significant relationship between these two carbon pools was found only in roots (Figure 6), where sucrose concentration was inversely correlated with starch concentration, indicating that starch depletion was associated with increased sucrose availability. This relationship was independent of the light treatment, suggesting that root starch mobilization contributed to sucrose supply under drought regardless of stem shading.

## 4 Discussion

Our data provide new insights into the role of stem photosynthesis in the dynamics of sugars and mannitol during drought and recovery in *F. ornus* . Stem shading induced an increase in xylem loss of hydraulic conductance and limited sucrose translocation to the roots. Moreover, our data suggest that osmotic adjustment required to face water limitation was supported by mannitol accumulation in the bark, also allowing for energy savings in terms of ATP consumed per equivalent osmolyte produced.

### 4.1 Stem Shading Reduces Glucose Levels in the Stem and Roots

Under well‐watered conditions, stem shading reduced glucose levels in bark, wood, and roots, indicating that stem photosynthesis contributes to local and downstream carbohydrate availability, even when water is not limiting, confirming its contribution to the whole‐plant carbon balance (Kocurek et al. 2020). The observed decrease in glucose levels likely reflects a reduction in local photosynthetic carbon fixation within bark chlorenchyma. Since hexoses‐P are the first product of photosynthesis, limited light availability directly constrains their formation and, consequently, the availability of sucrose. However, stem shading did not significantly affect the concentrations of the other sugars measured, including mannitol. This suggests that, under sufficient water supply, leaf‐level photosynthesis sufficiently supported the metabolic carbon daily demand and sugar translocation (Bhatla et al. 2018).

In our experimental conditions, fructose was below the detection limit in all tissues/organs analysed; however, we cannot exclude the presence of stored fructose at very low concentrations. In woody plants, fructose abundance varies substantially among species and organs (Hoch et al. 2003). Low fructose levels have already been reported in ash leaves (Kim et al. 2025), suggesting that in this species, fructose contributes minimally to the bulk of soluble NSC stored in the cells for energy needs and osmotic requirements. At the same time, the concurrent accumulation of mannitol suggests that fructose may be directly invested in mannitol synthesis (Figure S5). Importantly, this does not preclude a potential signalling role for fructose at low concentrations, as previously suggested (Jeandet et al. 2022).

In woody plants, roots can serve as the main carbon storage site after the translocation of photo‐assimilates produced in aboveground organs (Hennion et al. 2019). A recent analysis proposed that stem shading significantly reduces the photosynthate flux to heterotrophic organs, such as roots (Kocurek et al. 2020). In accordance with this hypothesis, in our experiment, glucose concentration in the roots of stem‐shaded plants was lower compared to light‐exposed plants.

### 4.2 Consequences of Stem Shading Under Drought Stress Conditions

In a previous study, a marked effect of stem shading on xylem vulnerability to embolism was detected (Natale, La Rocca, et al. 2023). The new analysis presented in this study highlighted that stem‐shaded plants showed a higher loss of hydraulic conductance at the same xylem pressure when compared to lighted plants (Figure 3), underscoring the extended benefits of stem photosynthesis in maintaining whole‐plant hydraulic functioning (Ávila‐Lovera et al. 2024). Indeed, as xylem tension increases during drought, embolism formation progressively impairs water transport and indirectly limits carbon assimilation (Zwieniecki and Secchi 2015; Konrad et al. 2019). Although direct in vivo evidence for xylem refilling mechanisms in *F. ornus* is still lacking, the similarity with other diffuse‐porous species suggests that NSC availability can play a key role in both embolism resistance and recovery (Tomasella, Casolo, et al. 2019). In this view, the photosynthesis of stem chloroplasts may contribute to providing energy and carbohydrates at a local scale (Teskey et al. 2008; De Baerdemaeker et al. 2017). In agreement with previous studies (Li et al. 2025; Pichierri et al. 2026), drought induced a progressive decrease in starch levels in bark and roots along with the progressive increase of PLC (Figure 4b). This relationship was independent of the light treatment, evidencing the priority for plants to mobilize starch reserves to face a drought event. Our data support the hypothesis that water shortage triggers the activation of starch degradation, providing soluble NSC (Pagliarani et al. 2019; Tsamir‐Rimon et al. 2021) that contribute to osmotic adjustment, turgor maintenance, and cell survival under water shortage.

In contrast, total soluble NSC did not show any alteration in response to changes in PLC (Figure 4a). This result highlights that bulk NSC pools are insufficient to explain drought‐induced hydraulic dysfunction and reinforces the importance of analysing individual sugar components and their compartmentation (Vuerich et al. 2023). Different soluble sugars may fulfil distinct physiological roles during drought and recovery, which are obscured when considering only total NSC concentrations.

### 4.3 Stem Shading Modifies Glucose, Sucrose, and Mannitol Interplay

In our experiment, sucrose levels increased in the bark during drought regardless of the light treatment. Bark parenchyma represents a critical interface between phloem and xylem (Tomasella et al. 2025) and may act as a ready‐to‐use storage site for sugar to be mobilized in case of rapid carbon need, such as loading sucrose into the apoplast for osmotic requirements (Pagliarani et al. 2019). We suggest that sucrose accumulation in bark results from increased sugar demand for osmotic adjustment combined with drought‐related impairment in phloem transport (Savi et al. 2019). Drought is known to increase phloem sap viscosity and reduce transport efficiency due to both physical constraints and anatomical changes (Dannoura et al. 2019), potentially leading to sucrose accumulation in stem regions (Salmon et al. 2019).

Upon re‐irrigation, sucrose levels declined (Figure 5a), consistent with the “phloem‐unloading” hypothesis, according to which sucrose mobilisation sustains hydraulic recovery (Tomasella et al. 2017).

Interestingly, stem shading significantly reduced sucrose levels in the roots during drought (Figure 5c), suggesting that prolonged stem shading limits sucrose translocation to the root sink when maintaining osmotic potential and cell turgor in above‐ground organs is most critical (Kaur et al. 2021). Our results indicate that starch is inversely correlated with sucrose in roots, suggesting its degradation to support the local sucrose synthesis (Figure 6). In contrast, lighted plants may benefit from carbon derived from stem photosynthesis, sustaining sucrose transport to roots despite increased phloem sap viscosity associated with drought (Kiorapostolou et al. 2020).

Consistent with observations in olive trees (Tsamir‐Rimon et al. 2021), mannitol levels increased in both stems and roots during drought, suggesting a key role for this molecule as an osmolyte alongside sucrose. The origin of mannitol in woody tissues remains debated, as it may be synthesised locally in stems (Davis and Loescher 1990) or translocated from leaves (Tsamir‐Rimon et al. 2021). Our data do not allow us to distinguish between these possibilities, although higher mannitol levels in shaded plants indicate that carbon allocation may shift towards polyols when stem photosynthesis is suppressed. Notably, unlike sucrose, mannitol levels remained high during the recovery phase, especially in shaded plants, suggesting a sustained role in osmotic regulation during rehydration (Zivcak et al. 2016).

The different fates of photoassimilates under stem shading are further reflected in the relationships between glucose‐sucrose and glucose‐mannitol in the bark. Glucose was positively related to sucrose, and this relationship did not differ between light treatments. In contrast, glucose and mannitol were positively related under stem shading, suggesting that, when bark photosynthesis is suppressed, a larger fraction of soluble carbon may be associated with mannitol accumulation rather than sucrose. A similar pattern emerged from the relationships between glucose and starch, suggesting that glucose released from starch degradation also contributes to maintaining high mannitol levels. From an energetic perspective, the accumulation of mannitol represents an efficient strategy for osmotic regulation in plants under stem shading. Compared with sucrose synthesis, the accumulation of mannitol may provide a more osmotically effective solution with lower energetic cost. Indeed, based on theoretical calculations, producing one molecule of sucrose from glucose and fructose halves the osmotic effect while consuming one UTP to produce UDP‐glucose (Figure S5). Conversely, the reconversion of mannitol into fructose‐P requires the consumption of one ATP, comparable to the ATP invested needed to phosphorylate hexoses following sucrose hydrolysis.

## 5 Conclusions

Our findings provide new insights into the dynamics of soluble carbohydrates under drought conditions combined with prolonged stem shading, highlighting the importance of distinguishing between different pools of sugars to fully understand the contribution of stem photosynthesis to drought. Indeed, in our experiment, sucrose showed the most pronounced changes across drought and recovery phases under different light treatments, while glucose appeared to be mostly redirected towards mannitol synthesis. We also demonstrate that stem shading significantly affects root solute content, likely through drought induced limitations of xylem flow and phloem unloading, resulting in reduced sucrose transport to sink organs (i.e., roots). Although often underrepresented, root processes provide valuable information on plant carbon allocation under stress.

Finally, our results emphasize the relevance of polyols such as mannitol as key solutes under low water availability. Given their direct interconversion with hexoses, polyols should be considered in studies of NSC and osmotic adjustment, particularly in families where their substantial concentration is established (e.g., Oleaceae, Apiaceae, Rubiaceae).

## Author Contributions

S.N., A.N., S.G., and V.C. designed the experiment; S.G. and S.N. performed the experiment and measurements, with help from V.C. and A.P.; S.N. and M.T. performed hydraulic measurements; S.G., S.N., A.P. and V.C. performed NSC extraction, analysis and data interpretation; S.G. and A.P. analysed the data; S.G. and V.C. wrote the manuscript, with contributions and revisions from all authors.

## Conflicts of Interest

The authors declare no conflicts of interest.

## Supporting information


**Table S1:** Outcomes of the linear models used to test the effect of light treatment, plant material and their interaction on glucose, maltose, sucrose and mannitol concentrations.
**Table S2:** Outcomes of the linear model used to assess the relationship between the percentage loss of conductivity (PLC) and xylem water potential (Ψ xyl) in different light treatments.
**Table S3:** Outcomes of the linear models used to test the effect of the percentage loss of conductivity (PLC), plant material and their interaction on soluble NSC and starch concentration.
**Table S4:** Outcomes of the linear models used to test the effect of water regime, light treatment and their interaction on sucrose and mannitol in bark, roots and wood.
**Figure S1:** Chlorenchyma tissue in *Fraxinus ornus* bark of two individuals grown in an open forest with Quercus pubescens and Ostrya carpinifolia in calcareous soil. Locality Avasinis, 200 m asl (Alesso, Friuli Venezia Giulia—46.2919 N, 13.0484 E). (A) 36 years; (B) 52 years. The age of the plants has been measured by counting the rings of a tree core.
**Figure S2:** Boxplots of chlorophyll a + b concentration in bark and wood after the stem shading. Yellow boxes: lighted (Li) plants; blue boxes: stem‐shaded (LS) plants. The *p*‐values of the explanatory variables (Water, W; Light Treatment, LT; and their interaction, W × LT) on plant parameters are also reported. *p < 0.05; **p < 0.01; ***p < 0.001.
**Figure S3:** Relationship between sucrose with PLC as measured in roots and on plants under different light treatments. Confidence interval (95%) is shown (light coloured areas). The *p*‐values of the explanatory variables (PLC; Light Treatment, LT) are also reported. *p < 0.05; **p < 0.01; ***p < 0.001.
**Figure S4:** Relationship between starch and mannitol concentration in bark, root and wood. Confidence interval (95%) is shown (light coloured areas). *p*‐values of the explanatory variables (PLC; Organ) are also reported. *p < 0.05; **p < 0.01; ***p < 0.001.
**Figure S5:** Relationship between glucose and sucrose, mannitol and starch concentration in bark as measured in lighted plants (yellow dots) and shaded plants (blue dots). Confidence intervals (95%) are shown (light coloured areas). The effects of the explanatory variables and their associated *p*‐values are also reported. *p < 0.05; **p < 0.01; ***p < 0.001.
**Figure S6:** Biochemical pathway from glucose 6‐phosphate produced with photosynthesis to mannitol and sucrose formation according to light conditions. UTP is evidenced in red to highlight the requirement of free energy.

## Data Availability

Data will be made available in a public Digital Repository after the paper has been accepted.
