# Supplementary material for: Stem Shading Promotes Mannitol Accumulation in the Bark of Fraxinus ornus and Prevents Sucrose Transport in Roots Under Drought
Source: Physiol Plant. 2026 Apr 20;178(2):e70894. doi: 10.1111/ppl.70894 (PMC13096247; doi:10.1111/ppl.70894)
Supplement: Supplementary file 1 — Table S1: Outcomes of the linear models used to test the effect of light treatment, plant material and their interaction on glucose, maltose, sucrose and mannitol concentrations. Table S2: Outcomes of the linear model used to assess the relationship between the percentage loss of conductivity (PLC) and xylem water potential (Ψ xyl) in different light treatments. Table S3: Outcomes of the linear models used to test the effect of the percentage loss of conductivity (PLC), plant material and their interaction on soluble NSC and starch concentration. Table S4: Outcomes of the linear models used to test the effect of water regime, light treatment and their interaction on sucrose and mannitol in bark, roots and wood. Figure S1: Chlorenchyma tissue in Fraxinus ornus bark of two individuals grown in an open forest with Quercus pubescens and Ostrya carpinifolia in calcareous soil. Locality Avasinis, 200 m asl (Alesso, Friuli Venezia Giulia—46.2919 N, 13.0484 E). (A) 36 years; (B) 52 years. The age of the plants has been measured by counting the rings of a tree core. Figure S2: Boxplots of chlorophyll a + b concentration in bark and wood after the stem shading. Yellow boxes: lighted (Li) plants; blue boxes: stem‐shaded (LS) plants. The p‐values of the explanatory variables (Water, W; Light Treatment, LT; and their interaction, W × LT) on plant parameters are also reported. *p < 0.05; **p < 0.01; ***p < 0.001. Figure S3: Relationship between sucrose with PLC as measured in roots and on plants under different light treatments. Confidence interval (95%) is shown (light coloured areas). The p‐values of the explanatory variables (PLC; Light Treatment, LT) are also reported. *p < 0.05; **p < 0.01; ***p < 0.001. Figure S4: Relationship between starch and mannitol concentration in bark, root and wood. Confidence interval (95%) is shown (light coloured areas). p‐values of the explanatory variables (PLC; Organ) are also reported. *p < 0.05; **p < 0.01; ***p < 0.001. Figure S5: Relation [file PPL-178-e70894-s001.docx]

**Table S1** Outcomes of the linear models used to test the effect of light treatment, plant material and their interaction on glucose, maltose, sucrose and mannitol concentrations

|  | *DF* | *F-value* | *P-value* |
| --- | --- | --- | --- |
| Glucose |  |  |  |
| Light Treatment | 1 | 18.8502 | < 0.0001 |
| Plant Material | 2 | 203.2132 | < 0.0001 |
| Light Treatment x Plant Material | 2 | 0.3981 | 0.6742 |
|  |  |  |  |
| Maltose |  |  |  |
| Light Treatment | 1 | 1.6543 | 0.2053 |
| Plant Material | 2 | 443.0567 | < 0.0001 |
| Light Treatment x Plant Material | 2 | 0.3325 | 0.7189 |
|  |  |  |  |
| Sucrose |  |  |  |
| Light Treatment | 1 | 0.0636 | 0.8022 |
| Plant Material | 2 | 483.0557 | < 0.0001 |
| Light Treatment x Plant Material | 2 | 0.5357 | 0.5894 |
|  |  |  |  |
| Mannitol |  |  |  |
| Light Treatment | 1 | 0.2124 | 0.6471 |
| Plant Material | 2 | 217.3302 | < 0.0001 |
| Light Treatment x Plant Material | 2 | 0.7476 | 0.4793 |

**Table S2** Outcomes of the linear model used to assess the relationship between the percentage loss of conductivity (PLC) and xylem water potential (Ψ xyl) in different light treatments.

|  | *DF* | *F-value* | *P-value* |
| --- | --- | --- | --- |
| PLC |  |  |  |
| Ψ xyl | 1 | 54.754 | < 0.0001 |
| Light treatment | 1 | 4.992 | 0.0328 * |
| Ψ xyl x Light treatmen | 1 | 3.202 | 0.0433 * |

**Table S3** Outcomes of the linear models used to test the effect of the percentage loss of conductivity (PLC), plant material and their interaction on soluble NSC and starch concentration.

|  | *DF* | *F-value* | *P-value* |
| --- | --- | --- | --- |
| Soluble NSC |  |  |  |
| PLC | 1 | 0.124 | 0.7254 |
| Plant material | 2 | 356.774 | < 0.0001 |
| PLC x Plant organ | 2 | 0.199 | 0.8202 |
|  |  |  |  |
| Starch |  |  |  |
| PLC | 1 | 13.471 | 0.0005 *** |
| Plant material | 2 | 25.707 | < 0.0001 |
| PLC x Plant organ | 2 | 3.513 | 0.0353 * |

**Table S4** Outcomes of the linear models used to test the effect of water regime, light treatment and their interaction on sucrose and mannitol in bark, roots and wood.

|  | *DF* | *F-value* | *P-value* |
| --- | --- | --- | --- |
| BARK |  |  |  |
|  |  |  |  |
| Sucrose |  |  |  |
| Water | 2 | 4.305 | 0.0218 * |
| Light | 1 | 0.068 | 0.7948 |
| Water x Light | 2 | 0.130 | 0.8785 |
|  |  |  |  |
| Mannitol |  |  |  |
| Water | 2 | 30.161 | < 0.0001 *** |
| Light | 1 | 3.903 | 0.056 . |
| Water x Light | 2 | 5.697 | 0.0075 ** |
|  |  |  |  |
| ROOT |  |  |  |
|  |  |  |  |
| Sucrose |  |  |  |
| Water | 2 | 59.461 | < 0.0001 *** |
| Light | 1 | 4.342 | 0.0475 * |
| Water x Light | 2 | 9.942 | 0.0007 *** |
|  |  |  |  |
| Mannitol |  |  |  |
| Water | 2 | 18.491 | < 0.0001*** |
| Light | 1 | 5.374 | 0.0270 * |
| Water x Light | 2 | 1.812 | 0.1796 |
|  |  |  |  |
| WOOD |  |  |  |
|  |  |  |  |
| Sucrose |  |  |  |
| Water | 2 | 18.860 | < 0.0001 *** |
| Light | 1 | 2.191 | 0.1489 |
| Water x Light | 2 | 8.741 | 0.0009 *** |
|  |  |  |  |
| Mannitol |  |  |  |
| Water | 2 | 29.795 | < 0.0001 *** |
| Light | 1 | 0.002 | 0.9648 |
| Water x Light | 2 | 3.029 | 0.0624 |


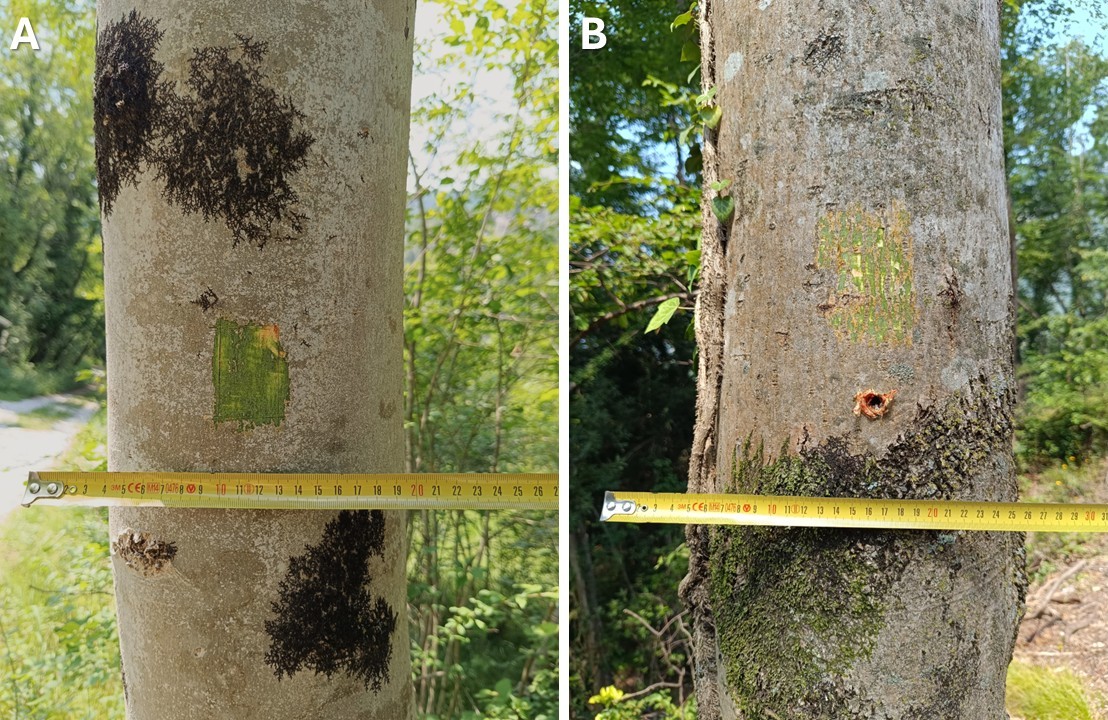


***Figure S1*** *Chlorenchyma tissue in Fraxinus ornus bark of two individuals grown in an open forest with Quercus pubescens and Ostrya carpinifolia in calcareous soil. Locality Avasinis, 200 m asl (Alesso, Friuli Venezia Giulia – 46.2919 N, 13.0484 E). A) 36 years; B) 52 years. The age of the plants has been measured by counting the rings of a tree core.*


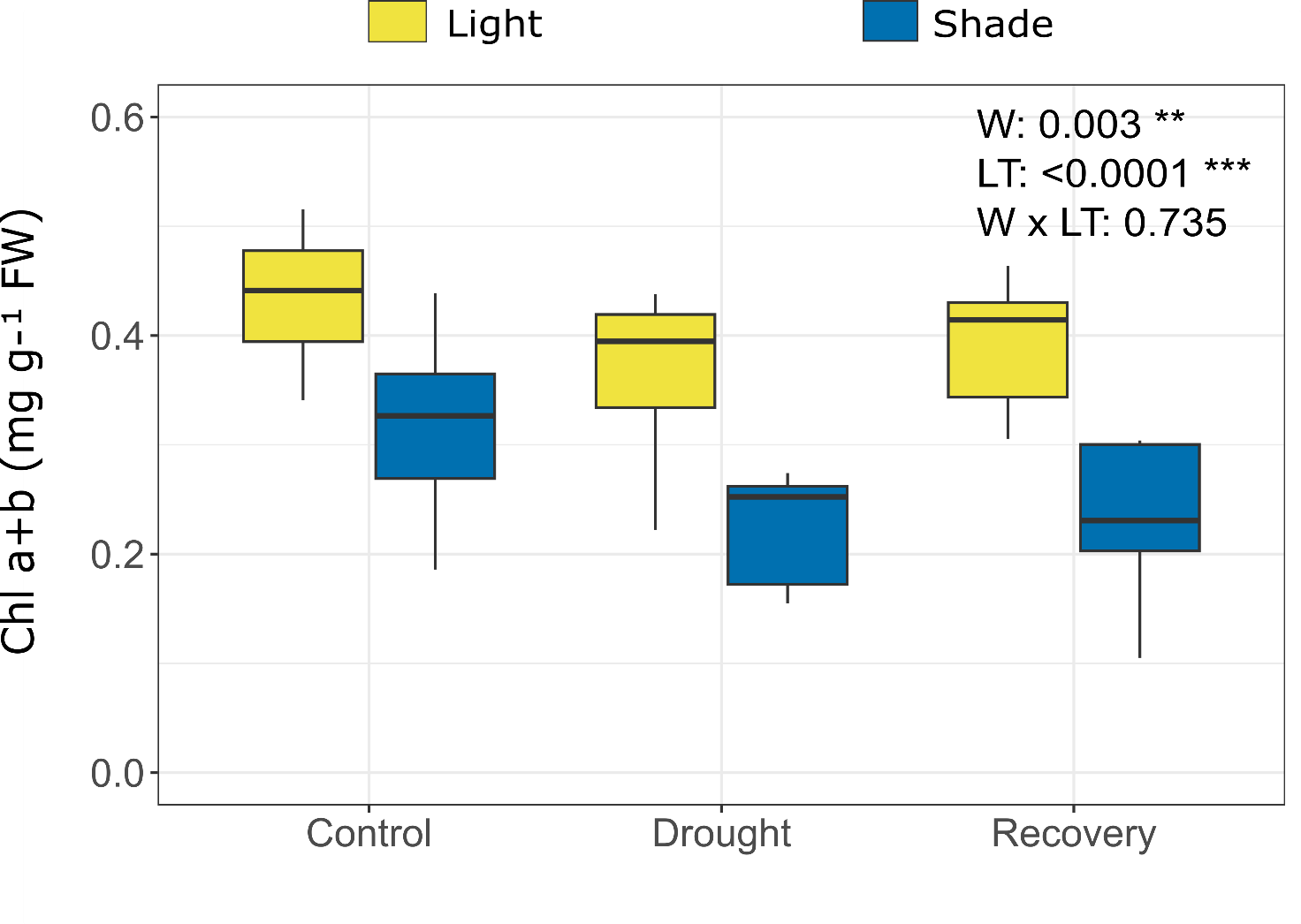


**Figure S2** *Boxplots of chlorophyll* a+b *concentration in bark and wood after the stem shading. Yellow boxes: lighted (Li) plants; blue boxes: stem-shaded (LS) plants. The* p-values *of the explanatory variables (Water, W; Light Treatment, LT; and their interaction, W x LT) on plant parameters are also reported. *= p < 0.05; ** = p < 0.01; ***= p < 0.001.*


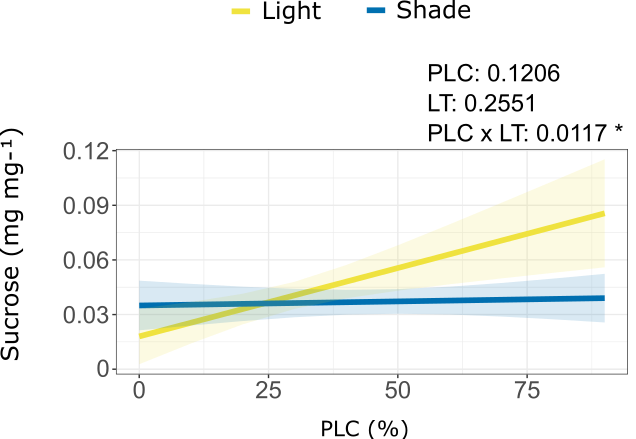


**Figure S3** Relationship between sucrose with PLC as measured in roots and on plants under different light treatments. Confidence interval (95%) is shown (light coloured areas). The p-values of the explanatory variables (PLC; Light Treatment, LT) are also reported. *= p < 0.05; ** = p < 0.01; ***= p < 0.001.


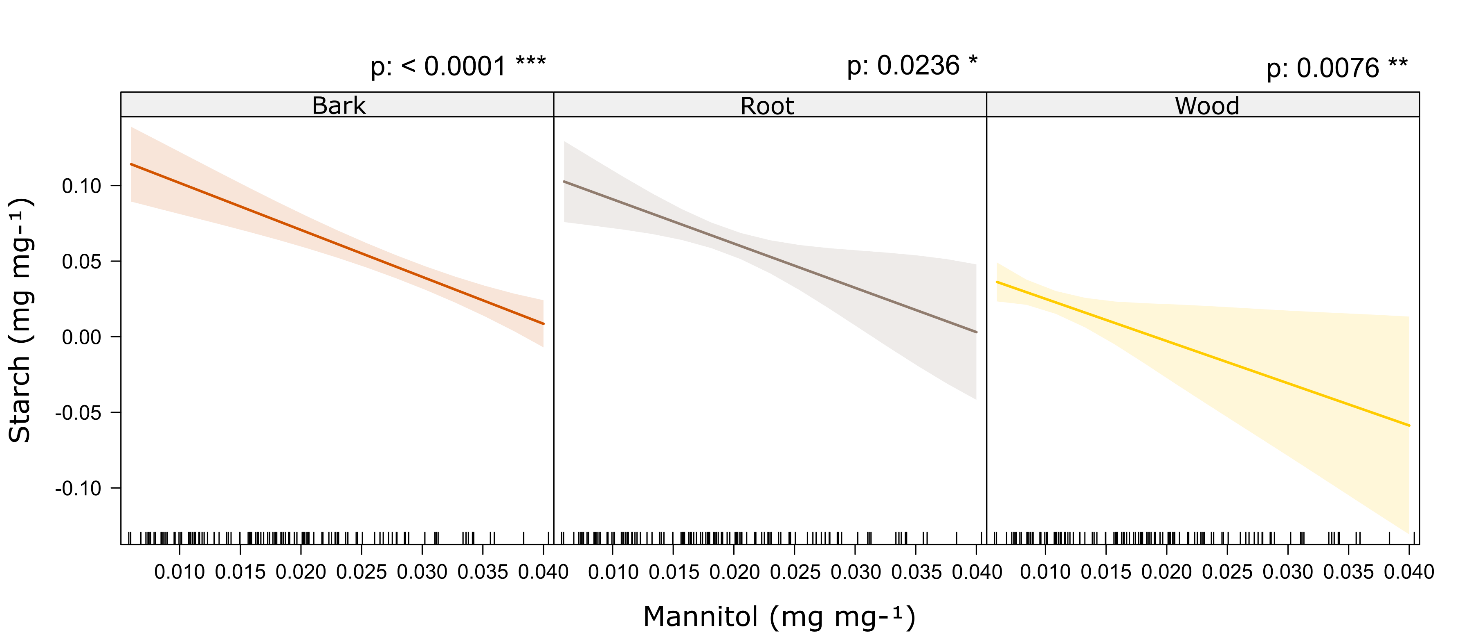


**Figure S4** Relationship between starch and mannitol concentration in bark, root and wood. Confidence interval (95%) is shown (light coloured areas). p-values of the explanatory variables (PLC; Organ) are also reported. *= p < 0.05; ** = p < 0.01; ***= p < 0.001.


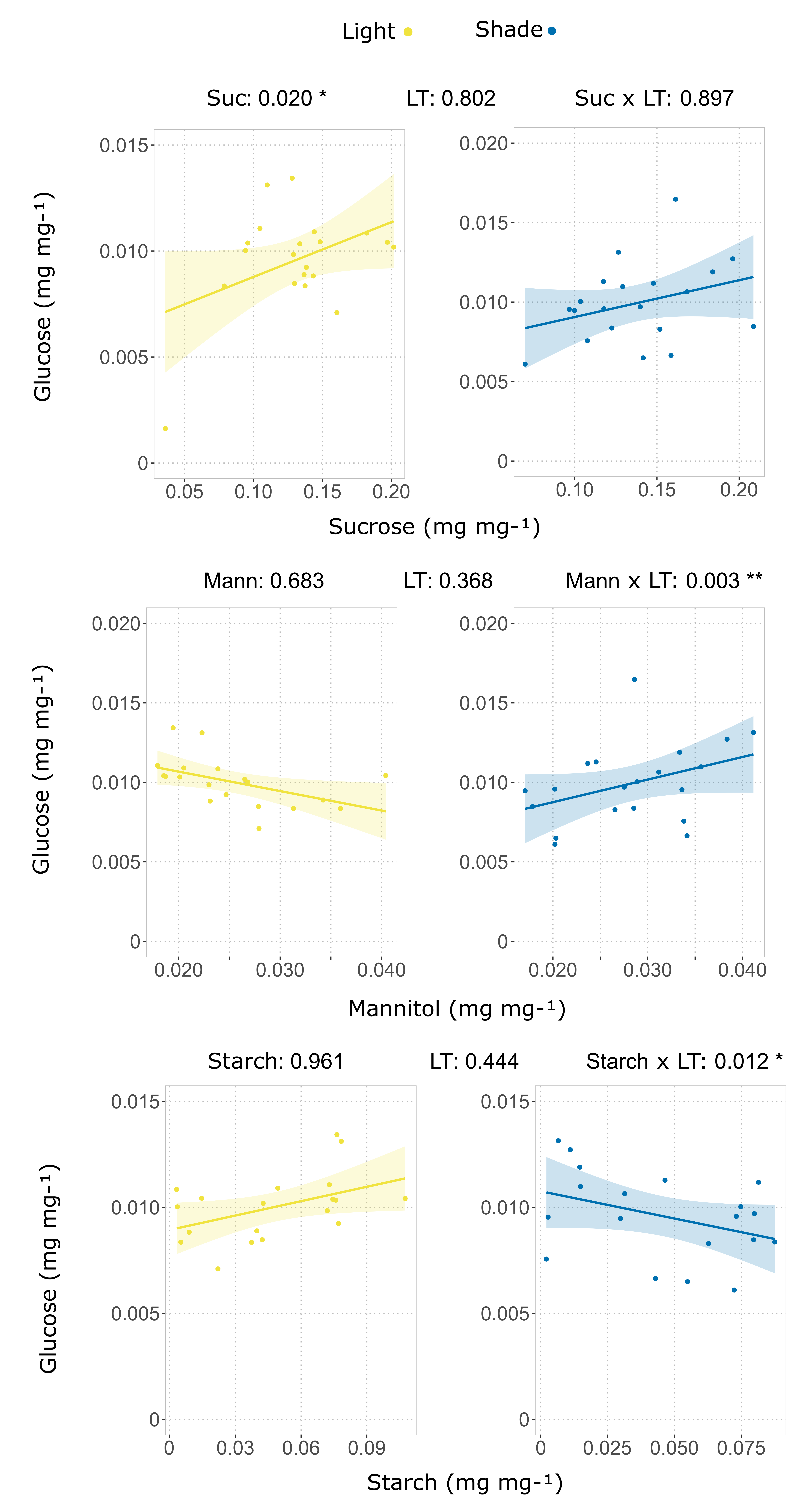


**Figure S5** Relationship between glucose and sucrose, mannitol, and starch concentration in bark as measured in lighted plants (yellow dots) and shaded plants (blue dots). Confidence intervals (95%) are shown (light coloured areas). The p-values of the explanatory variables and their associated p-values are also reported. *= p < 0.05; ** = p < 0.01; ***= p < 0.001.


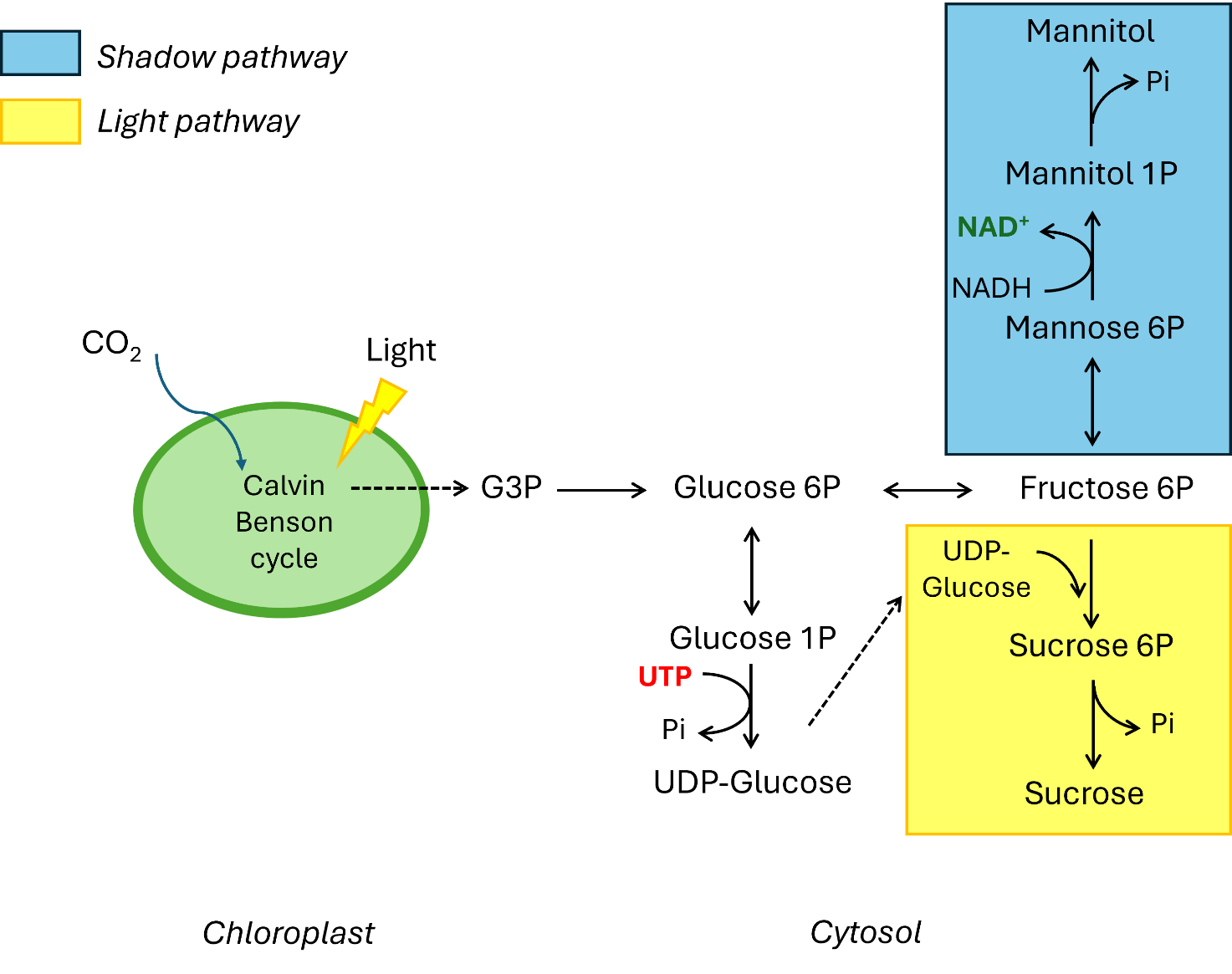


**Figure S6** Biochemical pathway from glucose 6-phosphate produced with photosynthesis to mannitol and sucrose formation according to light conditions. UTP is evidenced in red to highlight the requirement of free energy,
